# The mediating role of rumination in the relation between attentional bias towards thin female bodies and eating disorder symptomatology

**DOI:** 10.1371/journal.pone.0177870

**Published:** 2017-05-18

**Authors:** Laura Dondzilo, Elizabeth Rieger, Romina Palermo, Susan Byrne, Jason Bell

**Affiliations:** 1 School of Psychological Science, University of Western Australia, Perth, Western Australia, Australia; 2 ARC Centre of Excellence in Cognition and its Disorders, Macquarie University, Sydney, New South Wales, Australia; 3 Research School of Psychology, Australian National University, Canberra, Australian Capital Territory, Australia; University of Lincoln, UNITED KINGDOM

## Abstract

The present study sought to investigate the association between selective attentional processing of body images, rumination, and eating disorder symptoms in young women. Seventy-three undergraduate female students (ages 17–24) completed a modified dot-probe task to assess whether young women showed a differential attentional bias pattern towards thin and non-thin female bodies. Participants also completed self-report measures of eating disorder pathology. It was found that increased reports of dietary restraint and body dissatisfaction were associated with both greater attentional bias towards thin bodies and avoidance of non-thin bodies (as compared to neutral images), although the former relationship was stronger than the latter. The results suggest attentional vigilance to thin-ideal images plays a greater role in the potential development and/or maintenance of eating disorder symptoms, at least in a university sample of young women. Results also revealed that eating disorder-specific rumination mediated the relationship between attentional bias to thin ideal images and eating disorder symptoms. These findings build on existing research and theories, for example the impaired disengagement model of rumination, and have potential clinical applications such as specifically targeting ruminative and/or attentional processes in the prevention and/or treatment of eating disorder symptoms.

## Introduction

Exposure to advertisements glorifying the thin-ideal female body shape is pervasive. A meta-analysis of experimental and correlational studies revealed that media exposure to the thin-ideal is related to women’s vulnerability to body image disturbances and disordered eating behaviour [1]. More specifically, research has shown that women dissatisfied with their own bodies are particularly susceptible to negative affect and disordered eating after viewing thin-ideal stimuli [2–4]. Those with an attentional bias towards thin-ideal imagery may be especially vulnerable in an environment where such stimuli are ubiquitous. Theoretical and empirical evidence suggests that attentional biases towards body shape-related information, such as via thin-ideal media, may play an important role in the development and/or maintenance of eating disorder symptoms. In accordance with the transdiagnostic cognitive behavioural theory of eating disorders [5], these biases are hypothesised to arise from a dysfunctional scheme for self-evaluation that centres on the over-evaluation of eating, shape and weight and their control.

Empirical data show, an association between attentional biases in the processing of female body images and eating disorder symptoms [6–12]. When viewing body-related information of other females (including both whole bodies and body regions), it generally appears that women with elevated levels of eating disorder symptomatology selectively attend towards body stimuli connoting a thin physique and avoid non-thin body stimuli. For example, avoidance of body regions identified as causing dissatisfaction has been found in women with a high level of drive for thinness and body dissatisfaction [11]. Similarly, women high on eating disorder symptoms spent more time looking at attractive compared to unattractive body parts of other women [7]. These eye-tracking results have been replicated in a sample of overweight men and women viewing images of other men and women, respectively [13] and also in young women who were both dissatisfied with their own bodies and had a higher body mass index (BMI) [14]. Moreover, women with bulimia nervosa showed significantly longer fixations, relative to controls, when presented with images of female bodies that were thinner in comparison to the self [9].

Other studies have used a dot-probe methodology, in which participants are briefly presented with stimulus pairs (e.g., thin body image and a neutral image) and are required to discriminate a probe (e.g., single letter ‘p’ or ‘q’) that subsequently appears in the location of one of these two stimuli. Attentional bias is present when an individual is quicker to identify probes that replaced one stimulus type (e.g., thin body images) relative to other stimuli (e.g., neutral images). Glauert, Rhodes, Fink, and Grammer [15] found an automatic attentional bias to thin female bodies, compared to non-thin female bodies, in a non-clinical female sample. Further studies found that a greater attentional bias to thin bodies, relative to non-thin bodies, was associated with elevated levels of body dissatisfaction [6,8].

Although there is currently no research investigating whether attentional biases towards thin body images are causally involved in developing and/or exacerbating eating disorder symptoms, studies do show that attentional biases towards self-relevant body-related information may serve to trigger body dissatisfaction [16–18]. For example, an experimentally induced attentional bias towards young women’s own and self-defined unattractive body parts led to increased body dissatisfaction [16]. Similarly, studies by Smith and Rieger [17,18] showed that training young women to attend to shape/weight words connoting a large physique, with these words potentially processed as self-relevant, resulted in increased body dissatisfaction. However, induced body dissatisfaction did not lead to greater attentional biases [19]. In summary, individuals with eating disorder symptoms seem to attend to thin bodies/body regions of others (as compared to non-thin) and for themselves, focus on the non-thin regions. This attentional pattern would serve to exacerbate dissatisfaction with one’s own body. In considering these findings, it may be postulated that attentional biases in the processing of body shape imagery is a risk factor for developing eating disorder symptoms, such as body dissatisfaction.

The specific mechanism by which attentional bias triggers eating disorder symptoms remains unknown. One possibility is that a cognitive-affective process, such as eating disorder-specific rumination, serves to mediate the relationship between attentional bias and eating disorder symptoms. Several studies have found an association between eating disorder-specific rumination in the potential development and/or maintenance of general eating disorder symptoms [20–23], clinically significant levels of dietary restraint [23], and body dissatisfaction and anxiety [24]. Eating disorder-specific rumination is characterised by preoccupation with eating, shape and weight and their control [25,26] and has two distinct subcomponents: brooding and reflection [20,23]. Reflection is interpreted as active problem solving to alleviate one’s problems (e.g., “Write down what you think about your eating, weight and/or shape and analyse it”), while brooding reflects a passive comparison of one’s current situation with some ideal standard (e.g., “Why can’t I handle my eating better?”) [20,27]. Research has shown that these two ruminative components differentially predict eating disorder symptoms. For example, in samples of non-clinical females, only eating disorder-specific brooding was shown to associate with general eating disorder symptoms [21,23] and clinical levels of both dietary restriction and binge eating [23]. However, in a sample of females with a history of anorexia nervosa, only reflection on eating, shape and weight concerns showed an association with eating disorder symptoms [21]. These findings suggest that both ruminative brooding and reflection are relevant for the development and/or maintenance of eating disorder symptoms.

To the best of our knowledge, no research to date has investigated the relationship between attentional biases towards body shape-related information and eating disorder-specific rumination. However, it is plausible to hypothesise that such a relationship exists considering the existence of a relationship between attentional bias for negative information and depressive rumination [28–30]. Associations between attentional bias and depressive rumination remain evident even after statistically controlling for concurrent levels of depression [28,29]. Additionally, Joorman et al. [29] found that an attentional bias for images of sad faces was only significantly related to ruminative brooding, and not ruminative reflection. To account for such findings, Koster, De Lissnyder, Derakshan, and De Raedt [31] proposed the impaired disengagement hypothesis, which basically states that heightened rumination is due to difficulties in disengaging attention away from negative self-referent information. Recent empirical findings provide support for this theory by showing that greater rumination about depressive themes was associated with greater impairments in attentional disengagement from negative information, and not enhanced engagement with such information [32]. The theoretical and experimental evidence for an association between depressive rumination and attentional bias for depressogenic information suggests a potential relationship between an attentional bias towards body images and eating disorder-specific rumination. In turn, eating disorder-related rumination may serve to trigger eating disorder symptoms. Therefore, it is possible that eating-disorder-specific rumination may function as a mediator between attentional bias to body images and eating disorder symptoms.

The overall goal of the present study was to investigate the relationship between attentional biases to body images, eating disorder-specific rumination, and eating disorder symptoms in a university sample of young women. A female-only sample was recruited to maximize comparability with the findings from previous studies investigating selective attention for body images that have used predominantly female-only samples. In addition, gender differences have been found in attentional processing of body images. Specifically, research has shown that men with elevated levels of eating disorder symptomatology selectively attend towards images of muscular male bodies, as opposed to thin-ideal bodies [10]. The first aim was to investigate whether young women show a differential attentional pattern towards thin, and away from non-thin, female bodies, as compared to neutral images, using a modified dot probe task. It was predicted that young women would show an attentional bias towards thin bodies and attentional avoidance of non-thin bodies given that this is the general pattern of findings for studies using images of other women and non-clinical samples.

To date, no research has investigated which attentional mechanism (i.e., bias towards thin bodies versus bias away from non-thin bodies) plays a greater role in the potential maintenance and/or development of eating disorder symptomatology. As such, the second objective of the present study was to determine whether attentional bias towards thin body images is more strongly associated with the specific eating disorder symptoms of body dissatisfaction and dietary restraint, relative to attentional avoidance of non-thin body images. It was predicted that a greater attentional bias towards thin bodies and greater avoidance of non-thin bodies would be associated with greater reports of body dissatisfaction and dietary restraint. In addition, it was predicted that the relationships between attentional bias and both dietary restraint and body dissatisfaction would be stronger for bias towards thin versus non-thin bodies.

Finally, the current research aimed to examine whether eating disorder-specific rumination mediates the relationship between attentional bias to body images and both body dissatisfaction and dietary restraint. Based on theoretical and empirical research suggesting a relationship between valence-specific attentional bias and depressive rumination, together with emerging research suggesting an association between eating disorder-specific rumination and eating disorder symptoms, it was hypothesised that eating-disorder-specific rumination would mediate the relationship between attentional bias to female body shapes and the specific eating disorder symptoms of body dissatisfaction and dietary restraint.

## Method

### Participants

The present study was advertised to females-only on an online experiment system used by first-year undergraduate psychology students at the University of Western Australia. Seventy-three female undergraduate students agreed to participate in the study in exchange for course credit. Participants were between the ages of 17 and 24 (*M* = 18.59, *SD* = 1.28). The mean BMI was 21.84 (*SD* = 3.52, range = 15.92 to 34.13). Ethics approval to conduct this study was provided by the University of Western Australia Human Research Ethics Committee, and all participants provided written informed consent. Although some of the participants were 17 years old, they were deemed mature enough as university students to participate in this study.

### Self-report questionnaires

#### Ruminative response scale for eating disorders

The Ruminative Response Scale for Eating Disorders (RRS-ED; [20]) evaluates ruminative themes of eating, weight and shape, with two subscales: brooding and reflection. There are six items relating to brooding (e.g., “Think about a recent meal time wishing it had gone better”) and three items relating to reflection (e.g., “Write down what you think about your eating, weight and/or shape and analyse it”). Each of the nine items is assessed on a four-point Likert scale ranging from 1 (*almost never*) to 4 (*almost always*). A higher score indicates greater rumination. The RRS-ED demonstrates good internal consistency, test-retest reliability, and convergent and discriminant validity [20,21,23]. The Cronbach’s alpha for the Brooding and Reflection subscales in the present study was α = .91 and α = .84, respectively.

#### Dutch eating behavior questionnaire

The current study utilised the 10-item Dietary Restraint subscale of the Dutch Eating Behaviour Questionnaire (DEBQ; [33]), which assess the tendency to restrict food intake. Specifically, respondents rate on a five-point Likert scale, ranging from 1 (*never*) to 5 (*very often*), how often they engage in restrictive eating behaviours (e.g.,‘Do you try to eat less at mealtimes than you would like to eat?’). A higher score is indicative of more frequent dietary restraint. The DEBQ’s Restraint scale has demonstrated strong reliability and a supported factor structure [34]. The Cronbach’s alpha for the Dietary Restraint subscale in this sample was α = .96.

#### Body shape questionnaire

The Body Shape Questionnaire (BSQ; [35]**)** consists of 34 items assessing dissatisfaction regarding shape and weight. Participants rate how often they have experienced body shape/weight-related concerns (e.g., ‘‘Have you felt excessively large and rounded?”) over the past month according to a six-point Likert scale, ranging from 1 (*never*) to 4 (*always*). A higher BSQ score indicates greater body dissatisfaction. This questionnaire demonstrates high internal consistency among females, concurrent validity with other measures of body dissatisfaction, and the ability to discriminate between clinical and non-clinical individuals [35,36]. The Cronbach’s alpha for the total BSQ in the present study was α = .98.

### Stimuli

In the present study, 40 image pairs each comprising a body image of a positive (thin body) or negative (non-thin body) emotional valence and an image of a neutral emotional valence (abstract art) were required. An initial pool of 68 images of thin (*n* = 34) and non-thin (*n* = 34) female bodies were sourced from the internet. The bodies depicted in these images reflected relatively healthy representations of thin and non-thin bodies. For each body type there was an estimated small range of BMIs, with thin bodies approximated to be bordering on underweight, which is consistent with the ideal, and non-thin bodies likely to be at the upper end of the healthy to the lower end of the overweight BMI range. These images were cropped to focus on specific body regions, such as the abdominal region and thighs as they have been shown to cause high dissatisfaction in women [7,11,37].

Next, images were rated by a separate sample to prevent the participants taking part in the current study from having previous exposure to the body images. This pilot sample was comprised of 57 female first-year psychology undergraduates (aged 17–24) from the University of Western Australia. Specifically, participants assessed the valence and arousal of these images using the Self-Assessment Manakin (SAM) affective rating system [38], which is the same scale used by the International Affective Pictures System [39]. The SAM scale ranges from 0 (‘unpleasant’ for valence and ‘calm’ for arousal) to 9 (‘pleasant’ for valence and ‘excited’ for arousal). On the basis of these ratings, a final pool of 20 positively valenced thin body images (M = 6.19, SD = .70) and 20 negatively valenced non-thin body images (*M* = 3.84, *SD* = .72) were obtained. Thin and non-thin body images were matched for arousal (thin: *M* = 4.18, *SD* = .37; non-thin: *M* = 3.97, *SD* = .37) but differed significantly on valence. Stimuli also included cropped sections of abstract art, which were of a neutral valence (M = 4.77, SD = 0.23).

The images were colour JPEG computer files and approximately 11cm high and 7.3cm wide on the screen. The stimuli were presented on 1024 x 768 Dell CRT monitor running at 85Hz, driven by a Dell PC and using Matlab (2012b) to control stimulus presentations.

### Modified dot probe task

Participants were seated with their eyes approximately 125 cm from the monitor. Each trial commenced with participants attending to a fixation cross presented in the middle of a desktop computer screen. The purpose of the central fixation cross was to minimize the likelihood that participants were attending to either probe location at the start of each trial. The cross appeared for 1,000 ms and was then replaced by an image pair comprising one body image (i.e., either a thin or non-thin body) and one abstract image. One of the images was centred three degrees above the fixation cross, and the other image was centred three degrees below the fixation cross. After a fixed period of 500 ms the image pair was removed from the screen and the position occupied by one of the images was replaced by one of two probes, a letter “p” or “q”. Participants were instructed to identify the letter, by pressing the appropriate key on a keyboard, as quickly and accurately as possible. After a response was made, the next trial commenced.

The letter shown and the location of the probe was randomised with equal probability. The fact participants had to discriminate between two alternative probes was to ensure that attention was allocated to the probe location. In other words, these letters would be easily confusable in the instance that attention was not allocated to the probe location. Additionally, two stimuli pairing conditions were run: (1) thin bodies and abstract art and; (2) non-thin bodies and abstract art. The stimuli of interest (i.e., thin or non-thin bodies) were paired with abstract art images as they do not hold any body shape and/or weight-related properties. This pairing allows us to quantify the magnitude of attentional bias towards or away from each body type. The conditions were blocked with a random order and random individual trial order for each observer. There were 160 trials for each stimuli pairing combination, making a total of 320 trials. Additionally, there were nine practice trials preceding each stimuli pairing combination block.

### Procedure

To commence, participants completed the modified dot probe task. Subsequently, participants completed the self-report measures (i.e., RRS-ED, DEBQ, and BSQ). The questionnaires were presented online via Version 1.92+ of LimeSurvey [40], an advanced online survey system that was hosted on the University of Western Australia servers. Finally, participants’ height and weight was measured to allow the calculation of BMI.

### Statistical analyses

Data analyses were performed using SPSS. The criterion for statistical significance across all analyses was *p* < .05. In considering that probe discrimination reaction times (RTs) are indicative of attention to the task at hand *only* when probes are discriminated correctly, the data analysis for the modified dot probe task was based on RTs for correct trials only. One participant was removed from further analysis as more than 25% of their responses were incorrect. The remaining participants displayed very high accuracy on the modified dot probe task, averaging 95.07% overall. To correct for the potential effects of outlier reaction times (RTs), response latencies of <200 ms were excluded, which is in line with criteria used in previous studies [41,42]. To further eliminate outliers, RTs more than 2.5 standard deviations above each individual’s mean RT were also removed [43]. Mean probe RTs were used to calculate an attentional bias difference score for each of the stimulus pairing combinations (thin and non-thin) using the formula of MacLeod and Matthews [44], computed as [(upper probe/lower target—upper probe/upper target) + (lower probe/upper target—lower probe/lower target)/2]. These scores provided an index of the degree to which probe detection was facilitated or inhibited by images of thin and non-thin bodies. Positive values for the stimulus pairing combinations of body images and abstract art images reflect an attentional bias towards the body image.

For the main analyses, one sample t-tests were performed on the two attentional bias difference scores (thin and non-thin) to determine whether there were attentional biases towards or away from probes which replaced thin/non-thin body images, relative to neutral images. Next, bivariate correlations between the attentional bias difference scores and eating disorder-related correlates were tested using Pearson correlation analysis. Partial correlations, controlling for BMI, were also conducted.

In regards to the second aim, Lee and Preacher’s [45] test for differences between dependent correlations was performed to determine whether attentional bias towards thin bodies was more strongly correlated with the eating disorder-related symptomology, compared to attentional bias away from non-thin bodies.

**Fig 1:**
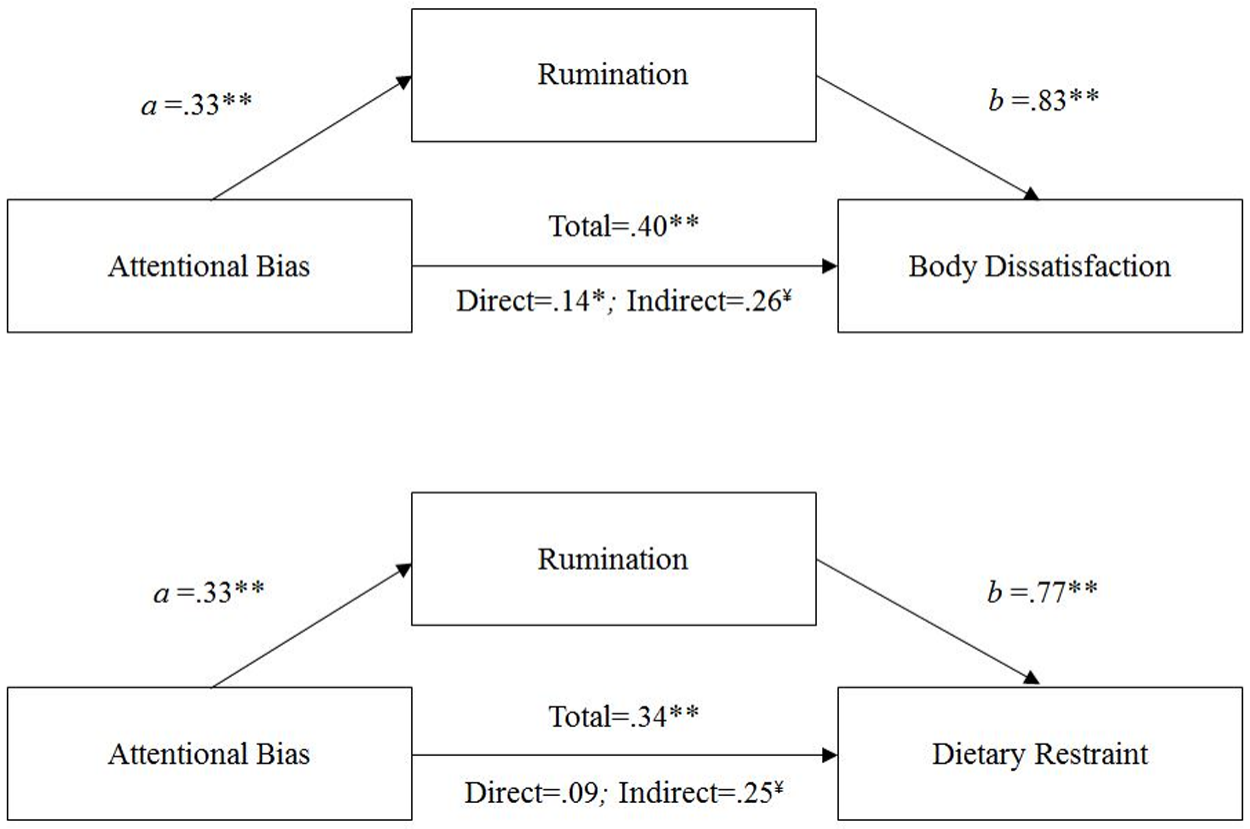
Mediation models representing the mediating effect of eating disorder-specific rumination on attentional bias to thin female bodies and body dissatisfaction (top) and dietary restraint (bottom). Paths *a* and *b* represent direct effects. All numbers are standardized OLS regression coefficients. BMI was included as a covariate. *p < .05; **p < .01. ¥–The 95% confidence intervals of the bias-corrected and accelerated estimate indicate a significant indirect effect.

In regards to the third aim, the indirect effect of attentional bias towards thin bodies on body dissatisfaction/dietary restraint through eating disorder-specific rumination was assessed using the bootstrapping procedure described by Preacher and Hayes [46]. The covariate of BMI was included in the models simultaneously with all other predictor variables. Bootstrapping generates an empirical approximation of the sampling distribution of the indirect effect, quantified as the product of the ordinary least-squares (OLS) regression coefficient estimating eating disorder-specific rumination from attentional bias (path *a* in Fig 1) and the OLS regression coefficient estimating body dissatisfaction/dietary restraint from eating disorder-specific rumination controlling for attentional bias (path *b* in Fig 1). In accordance with this procedure, 5000 bootstrap samples were drawn with replacement from the original sample to calculate bias-corrected and accelerated 95% confidence intervals for the indirect effect. Mediation was considered statistically significant (*p* < .05) if the 95% confidence intervals (CI) for the indirect effect did not include zero.

## Results

### Modified dot probe task

To determine whether the attentional bias difference scores for the thin and abstract image pairing (*M* = 2.35, *SD* = 20.94) and the non-thin and abstract image pairing (*M* = -6.27, *SD* = 22.84) were significantly different to zero, these scores were analysed by one sample *t*-tests. More specifically, since the attentional bias difference scores reflect the degree to which attention selectively moved to the location of thin/non-thin body images, compared to abstract art images, a value which differs significantly from zero is therefore indicative of a meaningful attentional bias.

Results showed that participants were significantly slower at detecting probes which replaced non-thin body images, compared to those replacing abstract art images, *t*(71) = -2.33; *p* = .02, indicating an attentional bias away from non-thin body images. On the other hand, the time taken for participants to detect probes which replaced thin body images, was not significantly different from the time to detect probes replacing abstract art images, *t*(71) = .95, *p* = .35, indicating no attentional bias towards or away from thin body images.

### Attentional bias and eating disorder-related correlates

Correlational analyses were performed to test the associations between thin and non-thin attentional bias difference scores and ruminative brooding and ruminative reflection on eating, shape and weight concerns, body dissatisfaction and dietary restraint. Prior to conducting the subsequent analyses, two outliers (defined as three standard deviations from the mean) were identified and removed, rendering the final sample size, *n* = 70.

Descriptive statistics and correlations between attentional bias difference scores and the self-report questionnaires are shown in Table 1. It can be seen that the degree of attentional bias to thin body images was significantly, positively, and moderately [47] associated with all eating disorder-related correlates. On the other hand, attentional bias to non-thin female bodies was significantly, negatively and moderately [47] associated with only dietary restraint and body dissatisfaction. All correlations remained significant after statistically controlling for participants’ BMI. Finally, results revealed that the relationships between attentional bias and specific eating disorder symptoms (i.e., dietary restraint and body dissatisfaction) was stronger for bias towards thin versus non-thin bodies (all *p* < .01).

**Table 1:** Bivariate correlations between AB difference scores for thin and non-thin bodies (ms) with eating disorder-related correlates (*n* = 70).

	Reflection	Brooding	Rest.	Body Diss.
**Mean (SD)**	4.77 (2.03)	12.64 (4.91)	26.86 (11.26)	95.87 (42.70)
**Thin**	.35**	.29*	.33*	.39**
**Non-thin**	-.20	4.91	-.30*	-.32**

Reflection, Ruminative Response Scale for Eating Disorders reflection subscale; Brooding, Ruminative Response Scale for Eating Disorders brooding subscale; Rest., Dutch Eating Behaviour Questionnaire dietary restraint subscale; Body Diss., Body Satisfaction Questionnaire;

*p < .05;

**p < .01

### Mediation analyses

To determine whether rumination on eating, shape, and weight concerns (i.e., the RRS-ED total score was used as both factors, brooding and reflection, equally mediated these relationships) functioned as a mediator between attentional biases to thin bodies and specific eating disorder symptoms (i.e., body dissatisfaction and dietary restraint), while statistically controlling for participants’ BMI, two mediation analyses were carried out using the bootstrapping procedure described by Preacher and Hayes [46].

Mediation analyses, which controlled for BMI, showed that rumination on eating, shape, and weight concerns did in fact mediate the relationship between attentional bias towards thin bodies and each of the following (see Fig 1): body dissatisfaction (*B* = .65, SE = .24, *p* = .007, 95% CI = .20, 1.15), and dietary restraint (*B* = .17, SE = .06, *p* = .005, 95% CI = .05, .30). As can be seen in Fig 1, the direct effect of attentional bias on body dissatisfaction was significant, suggesting partial mediation. However, the direct effect of attentional bias on dietary restraint was non-significant suggesting full mediation.

## Discussion

The current study was the first to show that eating disorder-specific rumination mediated the relationship between attentional bias to thin-ideal imagery and the specific eating disorder symptoms of body dissatisfaction and dietary restraint in a university sample of young women. These findings extend the literature demonstrating an association between depressive rumination and valence-specific attentional bias [28,30,32] to different types of rumination, namely, eating disorder-specific rumination. Also, while correlational in nature, the results are consistent with the suggestion that eating disorder-specific rumination contributes towards body dissatisfaction [24] and dietary restraint [23]. The mediational model accommodates various theories including the cognitive science perspective of impaired attentional disengagement leading to heightened rumination [31] and the novel process account of anorexia nervosa, which suggests that eating disorder-specific rumination may be associated with negative emotions and starvation-related body cues becoming less salient, thus driving dietary restriction [25,26].

Although the current findings cannot establish causal relationships, due to the cross-sectional design, it is possible that the relationship between rumination and attentional bias is bidirectional. For instance, it is possible that attentional biases arise from a schema centering on the overconcern with body shape and weight [48,49], which may be conceptualised as including eating disorder-specific rumination. This is an important question as it informs the development of novel treatment strategies for eating disorder patients. The incorporation of procedures designed to target ruminative and/or attentional processes is supported by preliminary evidence showing the effectiveness of body exposure in improving body satisfaction in females high in body dissatisfaction [50] and successful results for rumination focused interventions in individuals with medication-refractory residual depression, generalised anxiety disorder, and persistent persecutory delusions [51–53].

Results also showed that heightened attention to thin bodies, and enhanced attentional avoidance of non-thin bodies, were associated with greater reports of body dissatisfaction and dietary restraint, even after controlling for participants’ BMI. Ruminative reflection and ruminative brooding on eating and body shape concerns positively and significantly correlated with bias to thin bodies only. Further analyses revealed that dietary restraint, body dissatisfaction, and eating disorder-specific rumination correlated significantly more strongly with bias to thin bodies than with the avoidance of non-thin bodies. This may imply that attentional bias towards thin-ideal body shapes plays a more important role in the potential development of eating disorder symptoms. In turn, this identifies vigilance towards thin-ideal bodies as more maladaptive and a greater risk factor for developing an eating disorder, as opposed to the avoidance of non-thin bodies. These findings corroborate previous empirical research showing an association between bias to thin-ideal body shapes and eating disorder pathology [7,9,14]. The selective attentional pattern may represent maladaptive social comparison strategies [54] in the sense that young women with elevated eating disorder pathology engage in upward social comparison by comparing their body to those perceived as more attractive (i.e., attentional bias to thin bodies) and avoid those perceived as less attractive (i.e., attentional avoidance of non-thin bodies). This attentional pattern would serve to maintain dissatisfaction with one’s own body as it sustains unrealistic body ideals.

Finally, the current study revealed that overall, young women showed attentional avoidance of non-thin bodies, however, no attentional bias towards thin bodies. The absence of attentional bias towards thin bodies is somewhat contrary to the results of Glauert et al. [15] who found an attentional bias to thin female bodies in a non-clinical female sample. Several methodological differences between the two studies may account for these discrepant results. Firstly, the current study utilised photographs of real and clothed female bodies that were more ecologically valid compared to the computer-generated bodies used by Glauert et al. [15]. Furthermore, the current study measured attentional bias by pairing body images with neutral images, while Glauert et al. [15] paired thin and non-thin bodies, with the former pairing arguably more representative of a naturalistic setting.

Although the current study is unique in that it advanced on the theoretical and empirical research encompassing rumination-linked attentional biases in the domain of eating disorder symptoms, it has some limitations. Firstly, the cross-sectional data does not allow any definite conclusions to be reached about the relationship between attentional bias and eating disorder symptoms. Experimental studies, which manipulate attentional bias and/or rumination, are necessary to obtain evidence of causality. Moreover, to aid differentiation between the two attentional components (i.e., attentional orientation versus attentional disengagement) and to register biases in the later stages of attentional processing, future research could incorporate eye-tracking technology. A further direction for future research is to determine whether men also exhibit an association between attentional bias towards idealized male bodies (i.e., muscular and lean), eating disorder-specific rumination and eating disorder symptoms. Finally, it would be valuable to extend the current methodology to young women with a clinically diagnosed eating disorder as it cannot be assumed that findings on a community sample extend to clinical samples.

In summary, the current findings suggest that while young women generally avoid non-thin body shapes, those with a heightened attentional bias to thin-ideal imagery experience greater body dissatisfaction, dietary restraint, and eating disorder-specific rumination. Attentional vigilance towards thin-ideal bodies is therefore a potential risk factor for developing an eating disorder. Furthermore, the current study was the first to show that eating disorder-specific rumination functions as a mediator between selective attentional processing of thin-ideal imagery and eating disorder symptoms. This supports the relevance of eating disorder-specific rumination in linking shape-related attentional processes and eating disorder pathology. The current study therefore builds on existing theories relating to the role of attention and rumination in eating disorder pathology and may have clinical applications such as the potential integration of rumination or attentional-focused strategies for the prevention and treatment of eating disorders.
