# Correction: The mediating role of rumination in the relation between attentional bias towards thin female bodies and eating disorder symptomatology

**DOI:** 10.1371/journal.pone.0196143

**Published:** 2018-04-18

**Authors:** Laura Dondzilo, Elizabeth Rieger, Romina Palermo, Susan Byrne, Jason Bell

Reference 3 is incorrect. The correct reference is: Posavac HD, Posavac SS, Posavac EJ. Exposure to media images of female attractiveness and concern with body weight among young women. Sex Roles. 1998; 38: 187–201. doi:10.1023/A:1018729015490.

Reference 15 is incorrect. The correct reference is: Glauert R, Rhodes G, Fink B, Grammer K. Body dissatisfaction and attentional bias to thin bodies. Int J Eat Disord. 2009; 43: 42–49. doi:10.1002/eat.20663.

Reference 19 is incorrect. The correct reference is: Smith E, Rieger E. An investigation of the effect of body dissatisfaction on selective attention toward negative shape and weight-related information. Int J Eat Disord. 2010; 43: 358–364. doi:10.1002/eat.20703.

Refence 27 is incorrect. The correct reference is: Treynor W. Rumination reconsidered: A psychometric analysis. Cognit Ther Res. 2003; 27: 247–259. doi:10.1023/A:1023910315561.

There is an error in Table 1. Please see the correct table here.

**Table 1:** Bivariate correlations between AB difference scores for thin and non-thin bodies (ms) with eating disorder-related correlates (*n* = 70).

	Reflection	Brooding	Rest.	Body Diss.
**Mean (SD)**	4.77 (2.03)	12.64 (4.91)	26.86 (11.26)	95.87 (42.70)
**Thin**	.35**	.29*	.33*	.39**
**Non-thin**	-.20	-.18	-.30*	-.32**

Reflection, Ruminative Response Scale for Eating Disorders reflection subscale; Brooding, Ruminative Response Scale for Eating Disorders brooding subscale; Rest., Dutch Eating Behaviour Questionnaire dietary restraint subscale; Body Diss., Body Satisfaction Questionnaire;

*p < .05;

**p < .01.
